# Gold nanoparticle decorated titania for sustainable environmental remediation: green synthesis, enhanced surface adsorption and synergistic photocatalysis†

**DOI:** 10.1039/d0ra05607c

**Published:** 2020-08-11

**Authors:** Maheshika Perera, Lahiru A. Wijenayaka, Kumudu Siriwardana, Damayanthi Dahanayake, K. M. Nalin de Silva

**Affiliations:** Sri Lanka Institute of Nanotechnology (SLINTEC) Mahenwatte, Pitipana Homagama 10200 Sri Lanka lawij@ou.ac.lk; Department of Chemistry, The Open University of Sri Lanka Nawala 11222 Sri Lanka; Centre for Advanced Materials and Devices (CAMD), Department of Chemistry, University of Colombo Colombo 00300 Sri Lanka

## Abstract

Developing materials for efficient environmental remediation *via* cheap, nontoxic and environmentally benign routes remains a challenge for the scientific community. Here, a novel, facile, and green synthetic approach to prepare gold nanoparticle decorated TiO_2_ (Au/TiO_2_) nanocomposites for sustainable environmental remediation is reported. The synthesis involved only TiO_2_, metal precursor and green tea, obviating the need for any solvents and/or harsh chemical reducing or stabilizing agents, and was efficiently conducted at 50 °C, indicating the prominent sustainability of the novel synthetic approach. The synthesis indicated notable atom economy, akin to that observed in a typical chemical mediated synthesis while high-resolution transmission electron microscopy (HRTEM) findings suggest the presence of a pertinent decoration of spherical and homogeneous gold nanoparticles on the titania surface. Notably, the Au/TiO_2_ nanocomposite demonstrated appreciable stability during preparation, subsequent processing and prolonged storage. Further, the nanocomposite was found to have a superior adsorption capacity of 8185 mg g^−1^ towards methylene blue (MB) in solution using the Freundlich isotherm model, while the rate constants for the photocatalytic degradation of MB on the nanocomposite under UV irradiation indicated a 4.2-fold improvement compared to that of bare TiO_2_. Hence, this novel green synthesized Au/TiO_2_ nanocomposite shows promising potential for sustainable environmental remediation *via* efficient contaminant capture and subsequent synergistic photocatalysis.

## Abstract 2

Green synthesis of gold nanoparticle decorated titania for enhanced surface adsorption and synergistic photocatalysis.

## Graphical abstract

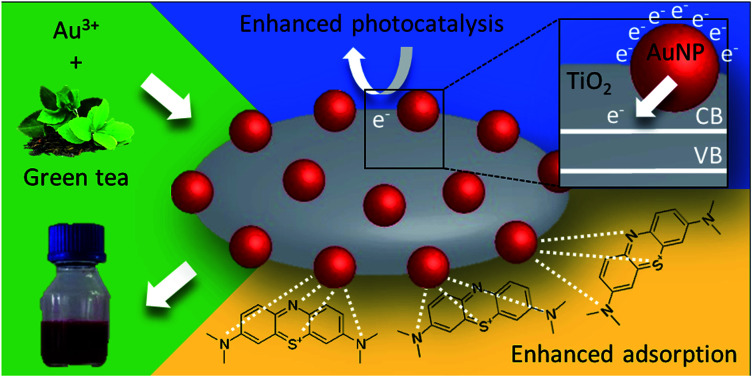

## Introduction

Among the many metal oxide semiconductor based photocatalysts, titanium dioxide (TiO_2_) is the most widely used, owing to its excellent optical transmittance, high refractive index and chemical stability, while concurrently being stable, nontoxic, and inexpensive.^1^ However, the wide band gap (∼3.2 eV) of TiO_2_ allows absorption of energy in the UV region of the solar spectrum, corresponding to only ∼5% of the solar spectrum.^2,3^ Additionally, TiO_2_ inherently suffers from the fast recombination of photogenerated excitons.^4^ Typically, for TiO_2_, the charge recombination time has been determined to be in the order of 10^−9^ s, while the chemical reaction time between photogenerated charges and any adsorbed species is 10^−8^ to 10^−3^ s.^4^ Hence, the fast recombination of charges hinder the photocatalytic reactivity of a species adsorbed on a TiO_2_ nanoparticle catalyst. TiO_2_, therefore, is far from being a perfect photocatalyst regardless of its widespread applications.

Nevertheless, there is ample room to further improve the photocatalytic activity of TiO_2_-based photocatalysts. According to recent findings, the localized surface plasmon resonance (LSPR) photosensitization or the electromagnetic field enhancement of catalytic material *via* the deposition of noble metal nanoparticles has been reported as an effective strategy in enhancing visible light absorption,^5–7^ thus leading to significantly improved photocatalysis. Notably, here the chemically inert metal is in a separate phase in interfacial contact with semiconducting titania, in contrast to what would result from doping.^8^ This strategy has been reported to be very effective in enhancing photocatalysis as the Fermi levels of noble metals are lower than that of TiO_2_. This allows the photo-excited electrons to be transferred from the conduction band (CB) to metal particles deposited on the TiO_2_ surface, while photo-generated valence band (VB) holes remain on the latter, thereby diminishing the possibility of electron–hole recombination.^4^

Noble metal nanoparticles such as gold (Au) and silver (Ag) have tremendous interest in photo reactions since their optical and electronic properties are highly tunable by changing the size, shape, and surface charge. As per recent literature, there are several reports on gold nanoparticles (AuNPs) loaded onto TiO_2_, where the immobilization of AuNPs on TiO_2_ produces visible light induced photocatalysis for the oxidation of organic substances in water.^9^ In their recent work, Guo *et al.* has demonstrated the development of Au@TiO_2_ plasmonic films with enhanced photocatalysis resulting from the surface plasmonic resonance of isolated AuNPs in TiO_2_ nanocavities and suppressed electron recombination.^10^

Li *et al.* have reported the synthesis of highly active mesoporous titania photocatalyst by embedding gold nanoparticles homogenously within the framework, where significantly improved photocatalytic activity is observed due to enhanced light absorption and improved quantum efficiency.^11^ Additionally, Bian *et al.* have demonstrated that the modification of TiO_2_ mesocrystals with AuNPs allows a strong photoelectrochemical response in the visible electromagnetic region. Diffuse reflectance spectroscopy measurements have demonstrated that a substantial portion of electrons injected from the AuNPs to TiO_2_ through the plasmonic excitation, anisotropically migrate through the TiO_2_ nanocrystal significantly hindering potential charge recombination.^7^

However, coupling of metal nanomaterials to a catalytic surface would typically utilize non-sustainable methods incorporating harsh or even hazardous chemicals and strong reaction conditions to allow efficient preparation of nanoparticles. Additionally, although TiO_2_ has been used as a white pigment from ancient times, while its safety to humans and environment is well established,^12^ reagents and conditions used for coupling of AuNPs onto it may hinder the intrinsic environmental and biological compatibility of titania. Hence, the development of composite materials for the efficient environmental remediation *via* cheap, nontoxic and environmentally benign routes remains a challenge to the scientific community.

Notably, biocompatibility of nanoparticles may be significantly enhanced *via* the use of biogenic synthetic pathways such as the use of microorganisms or plant-based extracts in the synthesis of nanoparticles.^13–15^ Although the incorporation of AuNPs to TiO_2_ thus enhancing the photocatalysis of the latter is well demonstrated, there are no previous accounts of sustainable green approaches to develop such eco-friendly and innocuous composite nanomaterials. Additionally, although many recent scientific efforts have focused on the preparation of novel nanomaterials that are proficient in environmental remediation, the focus has continued to be on the light mediated degradation of contaminants; or photocatalysis. Thus, other mechanisms by which efficient contaminant capture and removal can be conducted, such as surface adsorption which is facile and spontaneous, are relatively less exploited.^16–18^

Here, the preparation of a AuNP decorated TiO_2_ nanocomposite using an entirely green chemical synthesis approach is reported. AuNPs were synthesized by reducing HAuCl_4_ onto TiO_2_ particles using a green tea extract. Green tea here acts both as a reducing and stabilizing agent, thus obviating the need to utilize any auxiliary chemicals during the preparation and/or the application of the catalyst. To the best of our knowledge, there is no previous report on the synthesis of AuNP decorated TiO_2_ using an entirely green chemical approach for efficient environmental remediation through contaminant capture and photocatalysis.

The environmental remediation efficiency of the AuNP decorated TiO_2_ nanocomposite was investigated *via* (1) adsorption and (2) photodegradation of methylene blue (MB). As per the findings, the decoration of the TiO_2_ surface with AuNPs significantly increases the MB adsorption capacity of the catalyst while demonstrating an improved photocatalytic degradation rate constant for the same. Overall, this indicates the significant aptitude of the green synthesized Au/TiO_2_ nanocomposite towards proficient environmental remediation.

## Methods

### Materials and instruments

Titanium dioxide (TiO_2_) (anatase) and tetrachloroauric(iii) acid trihydrate (HAuCl_4_·3H_2_O) were purchased from Sigma Aldrich (USA) and Sisco Research Laboratories Pvt. Ltd. (India) respectively. Green tea extract was obtained from Fragrance Oils (International) Limited, UK. Methylene blue (MB) was obtained from HiMedia Laboratories (India). A Shimadzu UV-3600 UV-Vis-NIR spectrophotometer was used for all UV-visible spectroscopic and Localized Surface Plasmon Resonance (LSPR) measurements and centrifugation was performed using a Vision Scientific VS-15000N centrifuge. The hydrodynamic radii of the Au@Ag nanoparticles were determined *via* dynamic light scattering using a Malvern Nano-ZS zetasizer. Ultrapure water (conductivity < 0.05 μS cm^−1^) obtained from an Evoqua Water Technologies ultrapure water system was used in all synthesis, preparation, and experimental procedures.

### Synthesis of the Au/TiO_2_ nanocomposite

TiO_2_ powder was ground into fine particles and was suspended in ultrapure water to prepare a 250 ppm suspension. This was sonicated for 30 minutes to ensure the efficient dispersion of TiO_2_ particles resulting in a homogeneous milky suspension. Then, 25.0 mL of the as-prepared TiO_2_ suspension was heated to 50 °C in a water bath under continuous stirring. HAuCl_4_·3H_2_O (5 mM, 10.0 mL) and a solution containing the diluted green tea extract (25% v/v, 10.0 mL) were simultaneously added dropwise into the TiO_2_ suspension at a rate of 0.5 mL min^−1^ using a burette and a programmable syringe pump respectively. Here, the reduction of Au^3+^ ions was evidenced by a pink coloration of the originally milky suspension within few minutes after initiating the addition. The mixture was stirred for an additional 30 minutes after the complete addition of reactants to allow sufficient time for the reaction while maintaining the temperature at 50 °C. Then, the solution was removed from the water bath and was allowed to gradually cool to room temperature while stirring. The resulting Au/TiO_2_ nanocomposite was centrifuged at 7000 rpm for 10 minutes to remove the excess, unreacted reagents and was re-suspended in ultrapure water to ensure the original TiO_2_ concentration (*i.e.* 250 ppm). The as-purified Au/TiO_2_ nanocomposite was stored at 4 °C and was used as needed for characterization and analysis.

### Microscopic characterization

A drop of the Au/TiO_2_ nanocomposite dispersion was casted on a holey carbon copper grid and was allowed to dry at room temperature. Electron microscopic images were obtained using a high-resolution transmission electron microscope (HRTEM) (Jeol JEM 2100) operated at accelerating voltage of 200 kV. The elemental distribution on the sample area was analyzed using an energy dispersive X-ray (EDX) spectroscopy using a detector (EDAX-Ametek) attached to the HRTEM and the elemental composition was analyzed with elemental mapping in scanning transmission electron microscopic (STEM) mode at an accelerating voltage of 200 kV. Furthermore, the electron energy loss spectroscopic (EELS) image of gold nanoparticles was acquired with an EELS spectrometer (GATAN 963 EELS spectrometer) attached to the HRTEM with an energy resolution of 0.25 eV per channel in STEM spectral imaging mode.

### Dye adsorption on Au/TiO_2_

MB solutions of different known concentrations between 2–100 ppm were prepared. Each MB solution was mixed with a portion of the Au/TiO_2_ nanocomposite suspension (250 ppm) at a 1 : 1 volume ratio, followed by incubation in darkness for 1 hour under continuous stirring. The resulting solutions were centrifuged to precipitate all solid material, along with any MB adsorbed on Au/TiO_2_ nanocomposites. Then, the supernatant was analyzed using UV-visible spectroscopy to quantify the unadsorbed MB remaining in solution.

### Photocatalytic dye degradation on Au/TiO_2_

Equal volumes of Au/TiO_2_ nanocomposite suspension (250 ppm) and a standard MB solution were mixed and incubated in darkness for ∼1 hour under continuous stirring. Then, identical volumes of the MB–nanocomposite mixture were transferred into Petri dishes and the samples were irradiated with UV-C light for a known duration. The resulting solutions were then centrifuged at a high centrifugal force to precipitate all solid material, along with any adsorbed MB, and the supernatant was analyzed using UV-visible spectroscopy to quantify the unadsorbed and unphotoreacted MB remaining in solution. The experiment was repeated for different durations of UV irradiation and for bare TiO_2_.

## Results and discussion

Here, a novel method was developed for the facile modification catalytic titania with AuNPs *via* an entirely green synthetic approach mediated by green tea. The green tea extract contained a mixture of polyphenols such as catechin, epicatechin, epicatechin gallate, epigallocatechin, epigallocatechin gallate, epigallocatechin methylgallate, gallocatechin, and gallocatechin gallate, as well as caffeine, gallic acid, l-theanine, theobromine, and theophylline, similar to previous reports on green tea.^19,20^ The conditions used for the reduction of Au^3+^ into the AuNPs were previously optimized in a separate synthetic approach that was conducted in the absence of any TiO_2_ in the synthesis medium such as to produce significantly stable AuNPs of uniform size and morphology.

Notably, doping of the titania surface with gold nanoparticles has been extensively studied. According to Shibata *et al.* scanning transmission electron microscopy investigations along with density functional theory calculations have indicated that AuNPs preferentially attach to specific sites on the TiO_2_ surface forming an epitaxial and coherent heterointerface.^21^ Additionally, Matthey *et al.* have theoretically shown strong adhesion of gold clusters on TiO_2_.^22^ Hence, it was hypothesized that if the synthesis of AuNPs was conducted in the presence of TiO_2_ particles, the nucleation of AuNPs could occur on the TiO_2_ surface. Hence, eventually, a procedure was conducted (*vide supra*) using the optimized synthetic conditions to repeat the synthesis of AuNPs in the presence of TiO_2_ particles suspended in the synthetic medium, where Au^3+^ is reduced to form AuNPs that systematically deposit decorating the TiO_2_ surface.

The facile green synthetic approach adopted for Au/TiO_2_ nanocomposite here involved only the Au precursor and the green tea extract being concurrently added to a titania suspension, where the oxidation-prone polyphenols present in the extract are likely responsible for the reducing action.^16,23,24^ Typically, synthesis of AuNPs requires the presence of stabilizing agents to allow the produced nanoparticles to be stably dispersed in solution. Additionally, many synthetic approaches for AuNPs, including the well-known Turkevich method utilize harsh reaction conditions such as boiling temperatures in order for the chemical reactivity to prevail.^25^ In contrast, green tea here serves dual roles of a reducing and a stabilizing agent, hence precluding the need for any auxiliary chemical species, while the synthesis efficiently took place at 50 °C; a relatively mild temperature compared to the typical boiling conditions employed during the synthesis of AuNPs.

However, regardless of the absence of any strong reducing and/or stabilizing agent and at the mild temperature used, it was observed that the Au^3+^ reduced forming a pertinent nanoparticle decoration on the titania surface as evidenced by the clear reddish hue. This observation was akin to that expected in a chemical-mediated synthetic method, indicating the notable atom economy of the synthetic approach. Additionally, this color was stable during purification *via* centrifugation and under storage for prolonged durations, indicating the remarkable stability of the nanoparticles. Further, the Au/TiO_2_ nanocomposite could be efficiently recovered from the synthetic medium *via* centrifugation, leaving only a negligible trace of AuNPs in the supernatant, indicating the significant efficacy and atom economy in decorating the titania surface with AuNPs. The reddish milky suspension of particles obtained post-purification was stored until further use. Gravitational settling was visible in the sample with time and the suspension was homogenized prior to being used in the analysis as described below.

**Fig. 1:**
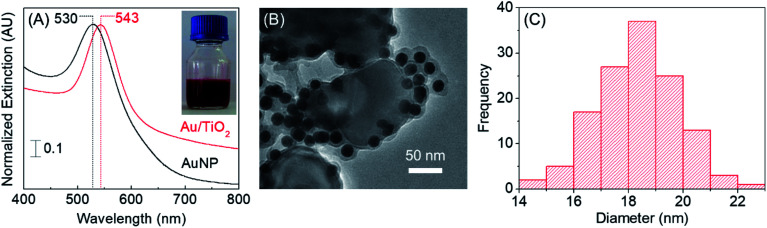
(A) Localized surface plasmon resonance (LSPR) spectra of bare AuNPs (black) and Au/TiO_2_ nanocomposite (red) while the inset shows a photograph of the Au/TiO_2_ nanocomposite, (B) representative TEM image of Au/TiO_2_ nanocomposite, and (C) AuNP particle size distribution (on the TiO_2_ surface) obtained *via* analyzing the TEM images.

The Localized Surface Plasmon Resonance (LSPR) spectra of as-synthesized Au/TiO_2_ nanocomposite and bare AuNPs prepared in an identical procedure, but in the absence of TiO_2_, are shown in Fig. 1(A), while the inset shows a photograph of the clear red colored Au/TiO_2_ nanocomposite. As can be seen, the wavelength of maximum extinction for the Au/TiO_2_ nanocomposite is ∼13 nm red shifted compared to bare AuNPs, being in good agreement with previous reports.^26^ The wavelength of maximum extinction for AuNPs have been previously reported to be dependent upon the size of the nanoparticles.^27^ Hence, it could be hypothesized that both the variation in the particle size of AuNPs as well as a change in the local refractive index of the AuNPs that takes place in the presence of TiO_2_, in combination are responsible for this shift in extinction maximum wavelength.

Further, the shift in the LSPR spectrum of Au/TiO_2_, specifically at the higher wavelengths can be attributable to the photoinduced charge separation at the plasmonic AuNP–TiO_2_ interface. Similar behavior has been previously reported for composite materials of Au and TiO_2_.^28,29^ Of note, such charge separation plays a prominent role in enhancing the quantum photocatalytic efficiency of a material.^30^ Hence, the LSPR spectrum of Au/TiO_2_ provided the initial evidence in support of the hypothesis on which the novel composite catalyst was developed herein.

Electron micrographs of the Au/TiO_2_ nanocomposite are shown in Fig. 1(B) and S1 included in the ESI.† These indicate the spherical and homogeneous AuNPs formed in the presence of TiO_2_, creating a pertinent decoration of AuNPs on the titania surface. Additionally, a coating likely resulting from the phytochemicals present in the green tea extract is visible around the AuNPs. The remarkable stability of the Au/TiO_2_ nanocomposite produced herein could be attributed to the presence of the above coating on the AuNPs, which is likely to reduce the propensity for any interaction driven particle coalescence. The average diameter of AuNPs was determined to be 18 ± 2 nm based on the measurements taken on randomly selected AuNPs (*n* > 100) present on TiO_2_ as shown in Fig. 1(C).

**Fig. 2:**
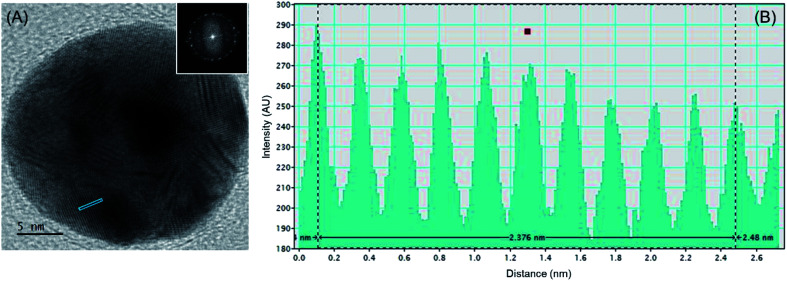
(A) HRTEM image of a single AuNP with the fast Fourier Transform (FFT) pattern indicated as the inset and (B) the intensity profile of the area indicated in blue on the nanoparticle.

Electron micrograph of a single AuNP is indicated in Fig. 2(A), with the fast Fourier Transform (FFT) pattern of the same is indicated as the inset. This indicates the near-perfect spherical geometry and the poly-crystallinity of the green-synthesized AuNPs on the titania surface. Additionally, Fig. 2(B) indicates the intensity profile of the area indicated in blue on the nanoparticle in the high-resolution transmission electron microscopic (HRTEM) image. Accordingly, the interatomic layer distance of gold in the green-synthesized AuNPs is found to be 2.4 Å. This indicates good agreement with the value previously reported for gold,^31^ thus offering credit to the sustainable approach developed herein.

**Fig. 3:**
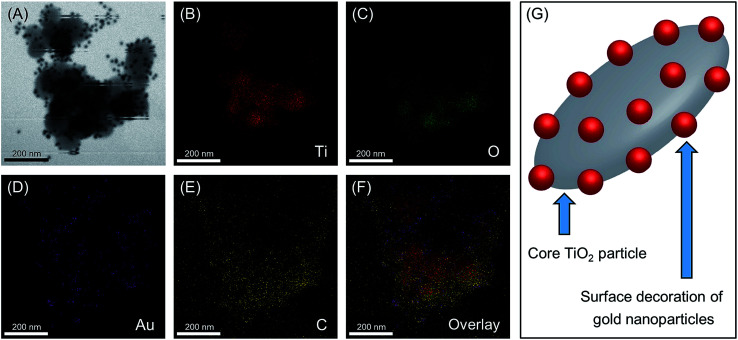
(A) Scanning transmission electron microscopic (STEM) image used for the energy dispersive X-ray (EDX) analysis of Au/TiO_2_ nanoparticles, and the elemental distribution maps for (B) Ti (red), (C) O (green), (D) Au (purple), (E) C (yellow), and (F) elemental map overlay within the area indicated on the STEM image, while panel (G) indicates an illustration of the gold nanoparticle decorated TiO_2_ structures as inferred by the EDX analysis.

The HRTEM images were analyzed further to confirm the presence and to investigate the distribution of AuNPs on the TiO_2_ surface. Fig. 3(A) shows the scanning transmission electron microscopic (STEM) image used for the energy dispersive X-ray spectroscopic (EDX) analysis of the Au/TiO_2_ nanoparticles, and panels (B), (C), (D), and (E) indicates the elemental distribution maps for Ti (red), O (green), Au (purple), and C (yellow) within the area indicated on the STEM image, while panel (F) indicates the overlay of the above elemental distribution maps. The EDX spectrum obtained for the above analysis is included in the ESI (Fig. S2†). The presence of a decoration of AuNP on TiO_2_ structures can be confirmed by the overlap of the elemental maps indicated in Fig. 3, where the results clearly show the localized presence of AuNPs on the TiO_2_ surface Hence, it is visible that the AuNPs are embedded on the TiO_2_ surface mimicking a plum-pudding or decorated composite architecture, as illustrated in Fig. 3(G) and as initially hypothesized here. Although comparatively smaller in number, the EDAX image also shows the presence of free AuNPs, other than those bound on TiO_2_. However, given that such free AuNPs observed during centrifugation was negligible (*vide supra*), the above observation is likely a consequence of sample preparation for HRTEM. Additionally, the presence of Au was confirmed with the EELS spectrum observed with Au M edge at 2206 eV, which is shown in Fig. S3 in ESI.†

Overall, the synthesis of these composite nanostructures could be conducted without the involvement of any chemical species other than the metal precursor and green tea, while maintaining mild temperatures throughout the procedure. Additionally, the process indicated notable atom economy, akin to that observed in a typical chemical mediated synthetic procedure. The resulting AuNPs indicate spherical and monodisperse nature while the Au/TiO_2_ nanocomposites indicate appreciable stability during processing as well as prolonged storage. From the detailed characterization conducted, it could be inferred that this green method results in a pertinent coating of spherical and homogeneous AuNPs on the titania surface *via* a facile and entirely sustainable approach. Collectively, therefore, the above factors offer notable credibility to the sustainability of the green synthetic approach presented herein.

With the strong electromagnetic coupling likely to occur at the interface between titania and surface-bound AuNPs, the developed Au/TiO_2_ nanocomposite can be used in wide variety of photo-mediated applications such as photocatalysis. Notably, the role of a photocatalyst is to enhance the rate of chemical reactivity under photo irradiation to facilitate any chemical degradation, resulting in improved photoremediation. However, as previously shown by Rodriguez *et al. via* synchrotron-based high-resolution photoemission and first-principles density-functional slab calculations, the deposition of gold nanoparticles on TiO_2_ produces a system with an extraordinary ability to adsorb and dissociate SO_2_, making Au/TiO_2_ much more chemically active than metallic gold or stoichiometric titania.^32^ Interactions between AuNPs and titania are likely to electronically perturb gold, making it more chemically active, while the same may facilitate the migration of O vacancies from the bulk to the surface of the oxide, enhancing the reactivity of titania.^32^

Therefore, the Au/TiO_2_ nanocomposite developed here would indicate two distinct and independent pathways for the efficient removal of contaminants from aqueous environments; namely (1) surface adsorption and (2) photocatalysis. Further, the proximity of the contaminants to the substrate, resulting *via* the adsorption of contaminants on the AuNP decorated titania, would facilitate efficient electron transfer thereby enhancing the photocatalytic activity. Hence, it was hypothesized that surface adsorption and photocatalysis in combination would produce a synergetic effect in the efficient removal of hazardous chemicals from contaminated waters. Accordingly, the ability of Au/TiO_2_ nanocomposite to be used in environmental remediation *via* enhanced surface adsorptions and ensuing photocatalysis was investigated using methylene blue (MB) as a model dye.

**Fig. 4:**
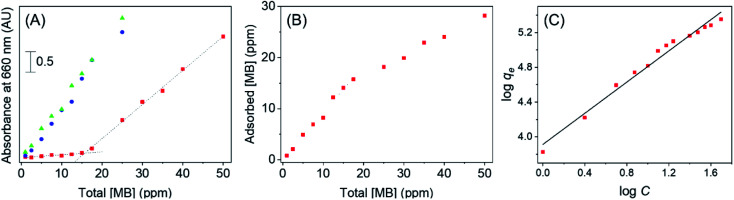
(A) Absorbance observed at varying total MB concentrations in the presence of TiO_2_ (blue circles), Au/TiO_2_ (red squares), and in the absence of any sorbent material (green triangles), (B) the hence calculated concentrations of adsorbed MB on Au/TiO_2_ at varying total MB concentrations, and (C) plot of ln *q*_e_*vs.* ln *C* obtained *via* the Freundlich isotherm modelling.

First, a dye adsorption study was conducted to quantify the extent of MB that can be effectively adsorbed onto the Au/TiO_2_ nanocomposite. A sample of bare TiO_2_ was analyzed in a similar procedure to determine any significant variations in adsorption capacity resulting from the AuNP decoration. Here, the UV-visible absorbance of free MB in solution was measured after complete dye adsorption onto the catalyst surface for both Au/TiO_2_ and bare TiO_2_. As seen from Fig. 4(A), the absorbance of free MB increased linearly in the presence of bare TiO_2_ with increasing initial MB concentration. This indicates that MB is only sparingly adsorbed onto the bare TiO_2_ surface. In fact, these values of absorbance at each MB concentration was very close to that of MB in the absence of any adsorbent, thus implying the significantly low or even negligible adsorption capacity of bare TiO_2_.

In contrast, for Au/TiO_2_, there was no significant absorbance in the supernatant until the MB concentration reached ∼17 ppm. This result suggest that MB will be completely adsorbed onto the Au/TiO_2_ catalytic surface at concentrations of MB below 17 ppm; a result attributable to the increased interactions arising due to the AuNPs on the surface of titania. Hence, the Au/TiO_2_ nanocomposite clearly indicates a significantly higher affinity towards MB compared to bare TiO_2_. Beyond the threshold concentration, the free MB concentration indicates a linear progression as a function of concentration, contrary to the surface saturation that one may expect to observe at high MB concentrations. This result was further analyzed by determining the concentration of adsorbed MB as indicated in Fig. 4(B). Accordingly, the adsorption onto Au/TiO_2_ shows a significant linear increase with the MB concentration up to ∼17 ppm, beyond which the adsorption continues to occur, but at a slower rate of increase.

Although it is typical for a sorbent surface to saturate upon the formation of a monolayer, the continual increase in adsorption suggests that the gold decoration on TiO_2_ promotes the formation of multi layers of MB on the sorbent surface. It is hypothesized that the initial rapid increase in MB adsorption on Au/TiO_2_ as seen in Fig. 4(B) is due to the monolayer formation, during which the dye molecules have the ability to adsorb onto the Au surface through the N or S atoms present in methylene blue. However, π–π stacking of MB molecules may allow further dye molecules to adsorb onto the catalyst surface, subsequent to monolayer formation, as have been previously observed with MB.^33^ Clearly, this behavior is attributable to notable enhancement in the overall adsorption capacity for MB on the Au/TiO_2_ nanocomposite developed here.

To better understand the adsorption process, the results indicated in Fig. 4(B) were fit to the Freundlich isotherm model which has the form;
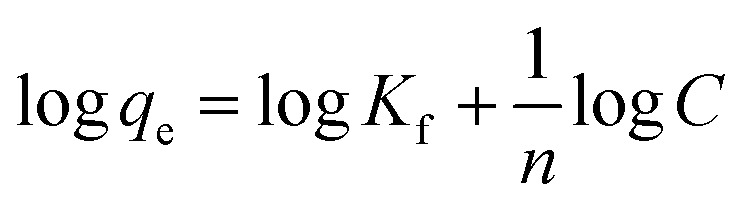
where *q*_e_ is the amount of MB adsorbed per gram of Au/TiO_2_ (mg g^−1^), *C* is the equilibrium MB concentration in solution (mg L^−1^), and *K*_F_ and *n* are the Freundlich constants which represent the adsorption capacity and the adsorption strength, respectively.^17^ The results are shown in Fig. 4(C), and as can be seen, the data indicates appreciable linearity (*R*^2^ = 0.9798) from which the values of *K*_F_ and *n* were found to be 8185 mg g^−1^ and 1.12 respectively. Of note, the Freundlich isotherm model assumes the possibility of multilayer adsorption as inferred previously from the results presented herein. Clearly, these findings imply that the adsorption capacity of the Au/TiO_2_ nanocomposite is superior to those previously reported for TiO_2_ modified specifically for the purpose of MB capture.^16,18,34,35^ Additionally, the value of *n* suggests favorable absorption on the substrate.^36^ Hence, the findings indicate that the Au/TiO_2_ nanocomposite indicates a superior adsorption capacity of 8185 mg g^−1^ towards MB in solution, demonstrating the remarkable potential of the green-synthesized nanocomposite towards environmental remediation *via* efficient contaminant capture.

The possibility of photosensitization of titania *via* gold by injection of electrons into the conduction band of the latter has been reported in previous literature. Tsukamoto *et al.* have confirmed the efficient transfer of electrons from photoactivated Au particles to titania by electron spin resonance (ESR) analysis of catalysts under visible-light irradiation.^37^ According to Su *et al.* the high photocatalytic activity could be attributed to the (1) plasmonic effect of gold nanoparticles, which enhances the visible light absorption, (2) increased surface area, (3) efficient charge separation, and (4) high carrier mobility of the titania.^38^ According to diffuse reflectance spectroscopic measurements reported by Bian *et al.*, a significant proportion of electrons are injected from the AuNPs to the titania through the surface plasmon excitation.^7^ Further, the close resemblance of the incident photon conversion efficiency spectrum to the plasmonic features of the Au/TiO_2_ system have suggested that the observed photocurrents originate from the plasmonic excitation of the AuNPs, indicating the prominent role of the plasmonic excitations of the AuNPs towards enhanced photocatalysis.^7^

**Fig. 5:**
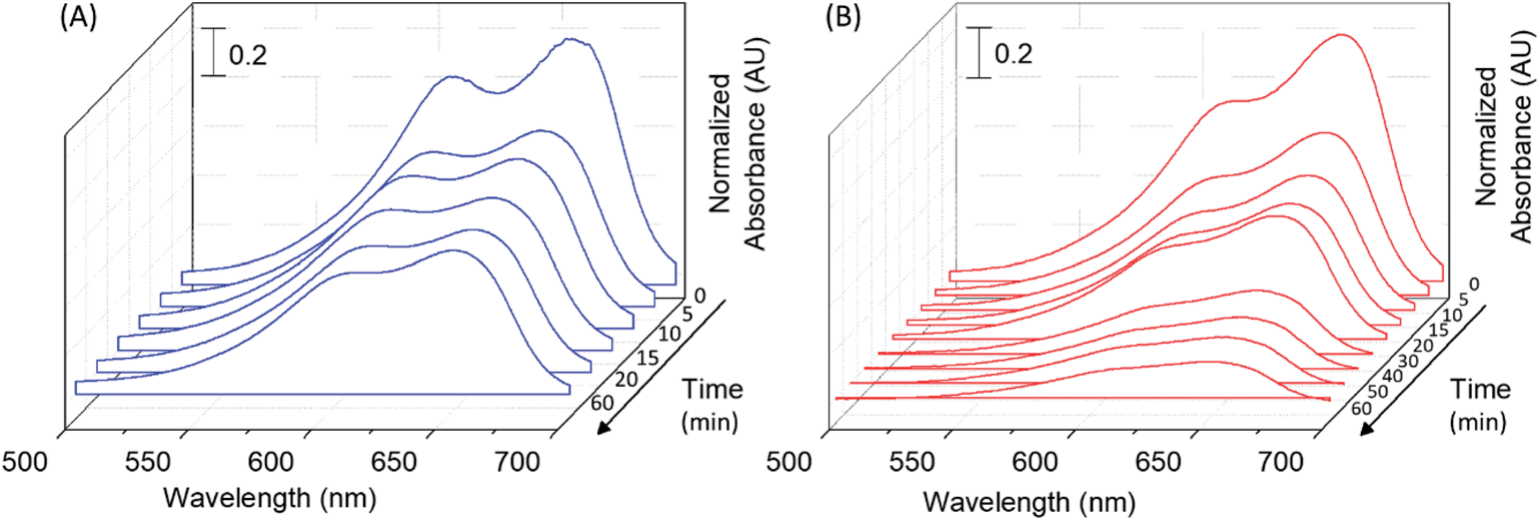
UV-visible spectra of free MB in solution, acquired after varying durations (0 to 60 min) of UV irradiation, for MB samples in the presence of (A) TiO_2_ and (B) Au/TiO_2_, indicating the photocatalytic degradation of MB.

Thus, the photocatalytic activity of fabricated nanocomposite was studied by measuring the rate of MB degradation under UV irradiation as indicated in Fig. 5. Here, Au/TiO_2_ nanocomposite and bare TiO_2_ samples, containing known concentrations of MB were irradiated with UV radiation for known durations. As can be seen on Fig. 5(A) and (B), the MB oxidizes *via* photoreactivity on both bare TiO_2_ as well as the Au/TiO_2_ nanocomposite, as evidenced by the decrease in absorption due to MB observed as a function of irradiation time. However, in the presence of Au/TiO_2_ nanocomposite, the MB solution turned clear within ∼30 min of UV irradiation, whereas, the blue color in the presence of bare TiO_2_ remained even after one hour of photo irradiation.

**Fig. 6:**
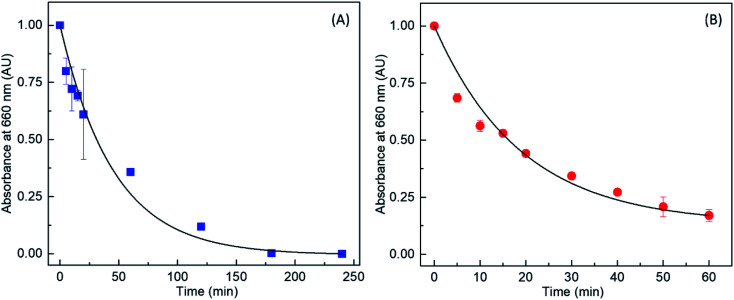
Variation in absorbance at 660 nm (due to free MB in solution) as a function of UV irradiation time, in the presence of (A) TiO_2_ and (B) Au/TiO_2_.

The results were further analyzed to obtain the time-dependent decrease in MB absorption and hence the rate constants for both bare TiO_2_ and the Au/TiO_2_ nanocomposite as shown in Fig. 6(A) and (B) respectively. Notably, it is observed that the photocatalytic rate constants of TiO_2_ and Au/TiO_2_ for the degradation of MB were 1.8 × 10^−2^ and 7.5 × 10^−2^ min^−1^, respectively, which indicates that the photocatalytic activity is enhanced by ∼4.2 times when TiO_2_ was decorated with AuNPs compared to bare TiO_2_. Similar enhancements in terms of MB degradation rate constants have been previously reported for Au and TiO_2_ composites.^39^

According to Quinones *et al.* the half-life of MB degradation over Au/Pd-modified TiO_2_ was 2.8 times smaller than pure TiO_2_ pure.^39^ Khalil *et al.* have reported the pseudo-first order reaction rate of MB over Au–TiO_2_ heterostructures to be 0.1570 min^−1^.^40^ Sangpour *et al.* have reported a degradation rate of 11.4 × 10^−3^ min^−1^ over a titania film containing AuNPs prepared by radio frequency reactive magnetron cosputtering.^41^ In their recent work, Yulizar *et al.* have reported that the photocatalytic activity of Au/TiO_2_ nanocomposite prepared *via* a plant extract mediated synthesis was 2.17 times higher than titania.^42^ However, the increase in reaction rate observed here *via* the surface decoration with AuNPs is significantly higher compared to those previously reported. Hence, this green synthesized Au/TiO_2_ nanocomposite shows promising potential for environmental remediation *via* efficient photocatalysis.

Hence, in summary, the Au/TiO_2_ nanocomposite developed here *via* the facile and sustainable green route indicates (1) prominent atom economy in preparation, (2) remarkable stability during preparation, processing, as well as prolonged storage, (3) superior adsorption capacity of 8185 mg g^−1^ for MB, demonstrating significant potential for efficient contaminant capture, and (4) significantly enhanced photocatalytic degradation rate of MB compared to bare TiO_2_. Additionally, given the widespread use of TiO_2_ based materials in environmental applications, and the facetious gravitational settling of this nanocomposite, efficient post-treatment removal of the material is possible from environmental or perhaps even other systems. Hence, collectively the improved capacity to capture as well as degrade MB in solution indicates that the nanocomposite has outstanding potential to serve in pronounced and sustainable environmental remediation.

## Conclusion

Here, the development of a novel, facile, and green chemical synthetic approach to prepare Au/TiO_2_ nanocomposites for sustainable environmental remediation is reported. The synthesis involved only the metal precursor and green tea, obviating the need for any solvents and/or harsh chemical reducing or stabilizing agents, and was conducted at mild conditions, allowing notable sustainability to prevail in the preparation of the novel catalyst. Experimental evidence indicated that the Au/TiO_2_ nanocomposite show a superior adsorption capacity of 8185 mg g^−1^ towards MB in solution, while the photocatalytic rate constants for the degradation of MB on the substrate indicated 4.2-fold improvement compared to bare TiO_2_. Adsorption capacity as well as the increase in reaction rate observed here were significantly higher compared to those previously reported, while there being no previous accounts of akin sustainable synthetic procedures for the preparation of composite nanomaterials of this nature. Hence, this novel green synthesized Au/TiO_2_ nanocomposite shows promising potential for environmental remediation *via* efficient contaminant capture, and subsequent photocatalysis.

## Conflicts of interest

There are no conflicts to declare.

## Supplementary Material

RA-010-D0RA05607C-s001
