# Supplementary material for: Gold nanoparticle decorated titania for sustainable environmental remediation: green synthesis, enhanced surface adsorption and synergistic photocatalysis
Source: RSC Adv. 2020 Aug 11;10(49):29594–602. doi: 10.1039/d0ra05607c (PMC9056003; doi:10.1039/d0ra05607c)
Supplement: RA-010-D0RA05607C-s001 [file RA-010-D0RA05607C-s001.pdf]

Electronic Supplementary Material (ESI) for RSC Advances.
This journal is © The Royal Society of Chemistry 2020

Electronic supplementary information

Gold Nanoparticle Decorated Titania for Sustainable Environmental Remediation: Green Synthesis, Enhanced Surface Adsorption and Synergistic Photocatalysis

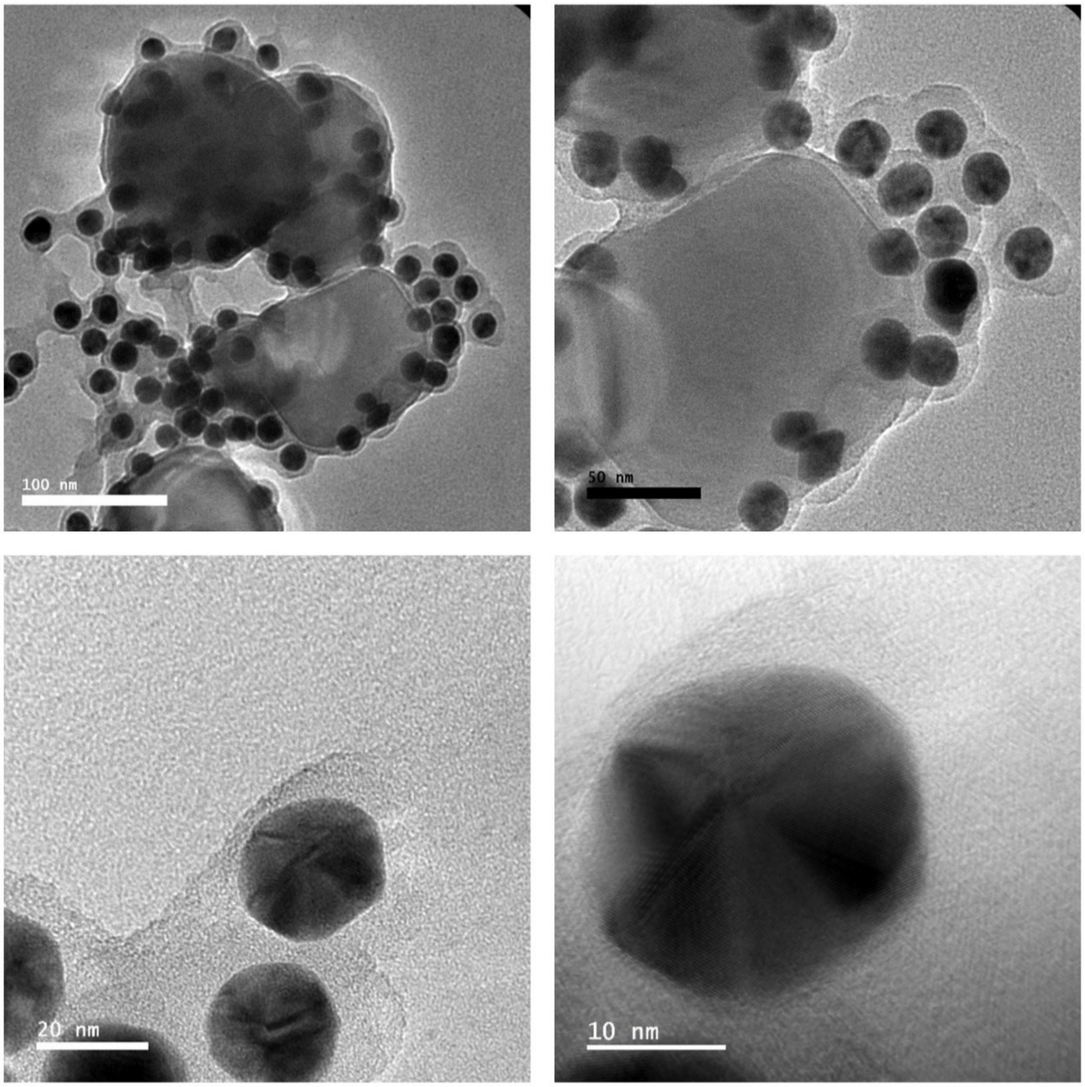

Figure S1. TEM images of Au/TiO_2 at varying levels of magnification

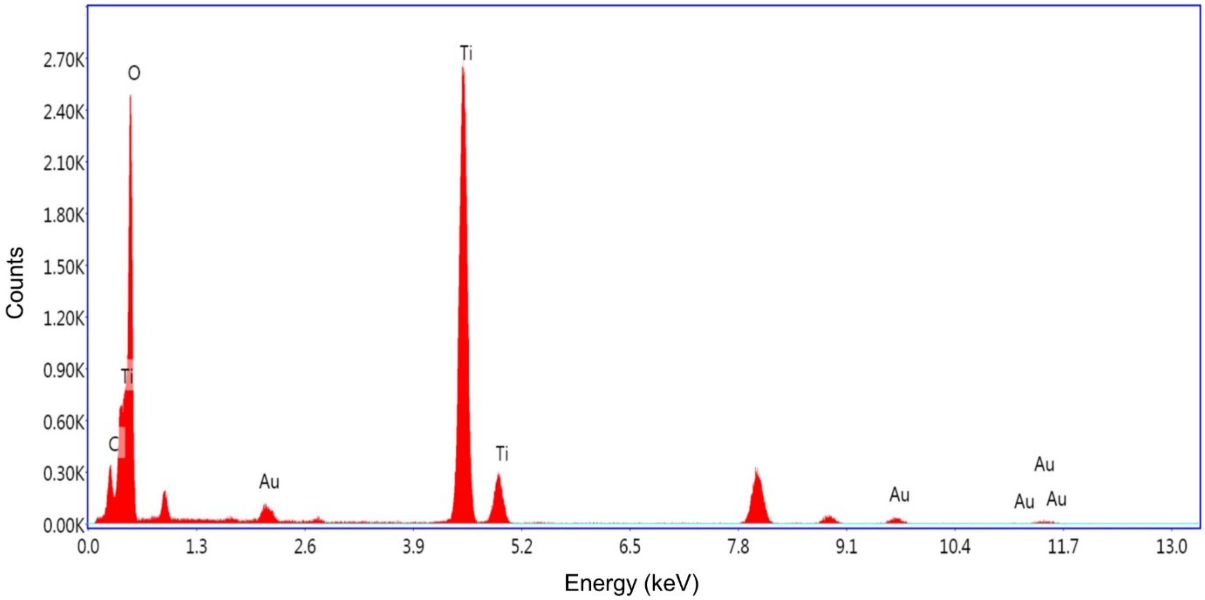

Figure S2. EDX spectrum for gold nanoparticles

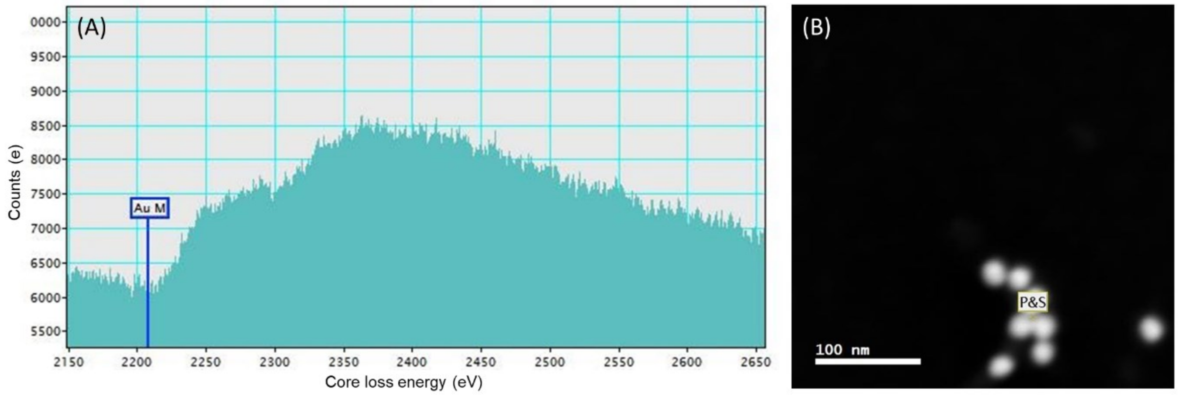

Figure S3. EELS spectrum with M edge of Au (a) for the position of gold nanoparticles marked as P & S in ADF image shown in (b)
